# AI-based smoke removal in robotic urooncologic surgery: a clinical pilot study

**DOI:** 10.1007/s11701-026-03951-z

**Published:** 2026-09-19

**Authors:** Leonhard Buck, Silja Janßen, Jakob Kohler, Julian Risch, Anton Moderegger, Daniar Osmonov, Jonas Jarczyk, Axel Merseburger, Kevin Köser, Philipp Nuhn, Severin Rodler

**Affiliations:** 1 Department of Urology, University Hospital Schleswig-Holstein, 24105 Kiel, Germany Campus Kiel,; 2 Kurt-Semm Center for Laparoscopic and Robotic Surgery, University Hospital Schleswig-Holstein, 24105 Kiel, Germany; 3 Department of Computer Science, Christian-Albrechts-University of Kiel, 24118 Kiel, Germany; 4 Department of Urology, University Hospital Schleswig-Holstein, Campus Lübeck, Lübeck, Germany 23538

**Keywords:** Surgical smoke removal, Endoscopic surgery, Artificial intelligence, Deep learning, 3D U-Net, Robotic surgery

## Abstract

Surgical smoke from electrocautery limits visibility during robot-assisted oncological surgery by obscuring tumor margins, blood vessels and anatomical landmarks. AI-based smoke removal demonstrated promising technical performance, structured clinical evaluation remains limited. This pilot study examined surgeons’ perceptions of a temporally consistent 3D U-Net applied to intraoperative smoke. Twenty urologists and residents retrospectively assessed ten pairs of endoscopic videos offline, showing original and AI-processed clips. A questionnaire evaluated general impressions (seven items, five-point Likert scale), direct comparisons across four image dimensions, open-ended feedback and overall utility. Visibility of anatomical details and depth perception were rated equal to the smoke-affected original by 75% of participants and slightly better by 25%. Color fidelity was rated equal by 85% and slightly better by 15%. None rated these dimensions worse (0/20, exact 95% CI 0 to 16.8%), and no participant selected the highest rating. Image stability was rated worse by 20%. Half disagreed that the processed footage appeared clearer. Overall utility was rated positively by 40%, neutrally by 35% and negatively by 25%. In this small, unblinded, single-institution retrospective pilot study, AI processing was not associated with a perceived worsening of anatomical detail, depth perception or color fidelity. However, confidence intervals remain wide and the study was not designed to establish equivalence. Most participants perceived no improvement in image clarity or orientation support. Artifact reduction, real-time performance and blinded multicenter evaluation are required before clinical use.

**Supplementary Information:**

The online version contains supplementary material available at https://doi.org/10.1007/s11701-026-03951-z.

## Introduction

Robot-assisted laparoscopic surgery has become the standard of care for a wide range of urooncologic procedures, including radical prostatectomy, partial nephrectomy and increasingly radical cystectomy [1–4]. Surgeons rely exclusively on the endoscopic video feed for navigation, tissue dissection and oncological decision-making. Therefore, precise visualization of anatomical structures, including tumor margins, dissection planes and vascular anatomy, is essential to achieving oncological radicality while minimizing intraoperative complications [5]. Energy devices generate surgical smoke through thermal tissue vaporization, which can impair endoscopic visibility [6]. In robot-assisted procedures, where surgeons lack direct tactile feedback and depend entirely on visual information, even brief episodes of smoke-induced visual impairment during critical steps may compromise surgical precision, delay decision-making and increase procedural complexity [7].

Mechanical smoke evacuation does not fully prevent transient obscuration and may affect pneumoperitoneal pressure [8]. The confined nature of pelvic and retroperitoneal spaces, coupled with the growing use of low-pressure pneumoperitoneum [9], further underscores the importance of effective smoke clearance.

Artificial Intelligence (AI)-based image enhancement aims to restore visual clarity directly within the endoscopic video stream. Several approaches have achieved promising performance on established image quality measures such as the Structural Similarity Index Measure (SSIM) and Peak Signal to Noise Ratio (PSNR). These approaches include dark channel prior (DCP) methods [10], Swin transformer [11], Generative Cooperative Networks [12] and supervised U-Net architectures [13, 14]. Most existing methods analyze individual frames separately and do not utilize the temporal information available across video sequences. This can lead to inconsistencies between consecutive frames that appear as flickering and may be as disruptive to the surgeon’s view as the smoke itself [15]. Janßen et al. addressed this issue by using a 3D U-Net to jointly process five consecutive frames. They achieved lower warping error and a higher SSIM than a framewise 2D [17]. However, standard image quality metrics do not reflect all aspects that matter to urooncologic surgeons, including tissue color preservation, anatomical boundary delineation, depth perception and the absence of distracting artifacts. Consequently, the clinical utility and acceptance of AI-based smoke removal remain largely unexplored.

This pilot study was conducted to address this gap. This study focuses on a different question: whether urologic surgeons would find this model’s output acceptable when applied to authentic intraoperative smoke rather than synthetic smoke. To our knowledge, this is one of the first structured clinical evaluations of a temporally consistent 3D U-Net approach for smoke removal conducted with urologic surgeons and residents. The primary aim was to investigate whether AI processing of robotic urooncologic surgery videos affects the perceived quality of clinically relevant image characteristics, such as visibility of anatomical details, depth perception and color fidelity. Secondary aims included evaluating perceived image clarity, the presence of artifacts and the overall clinical usefulness of smoke removal in specific procedural situations.

## Materials and methods

### Study design

This single-institution, questionnaire-based pilot study was conducted at the Department of Urology at the University Hospital Schleswig-Holstein (UKSH) Campus Kiel and Campus Lübeck from March - July 2026.

All evaluations were conducted retrospectively and offline using video footage selected from archived recordings of completed procedures. Participants reviewed the footage outside the operating room. No intraoperative or real-time evaluations were performed. They were then asked to provide structured ratings via a questionnaire designed for this purpose. This questionnaire-based study was designed and reported in accordance with the Consensus-Based Checklist for Reporting of Survey Studies (CROSS) [16]. The completed checklist is provided in the Supplementary Materials (Table S1). During the preparation of this work the authors used Claude (Anthropic) solely for language editing (grammar, spelling and phrasing). After using this tool/service, the authors reviewed and edited the content as needed and take full responsibility for the content of the published article.

### AI system under evaluation

**Fig. 1:**
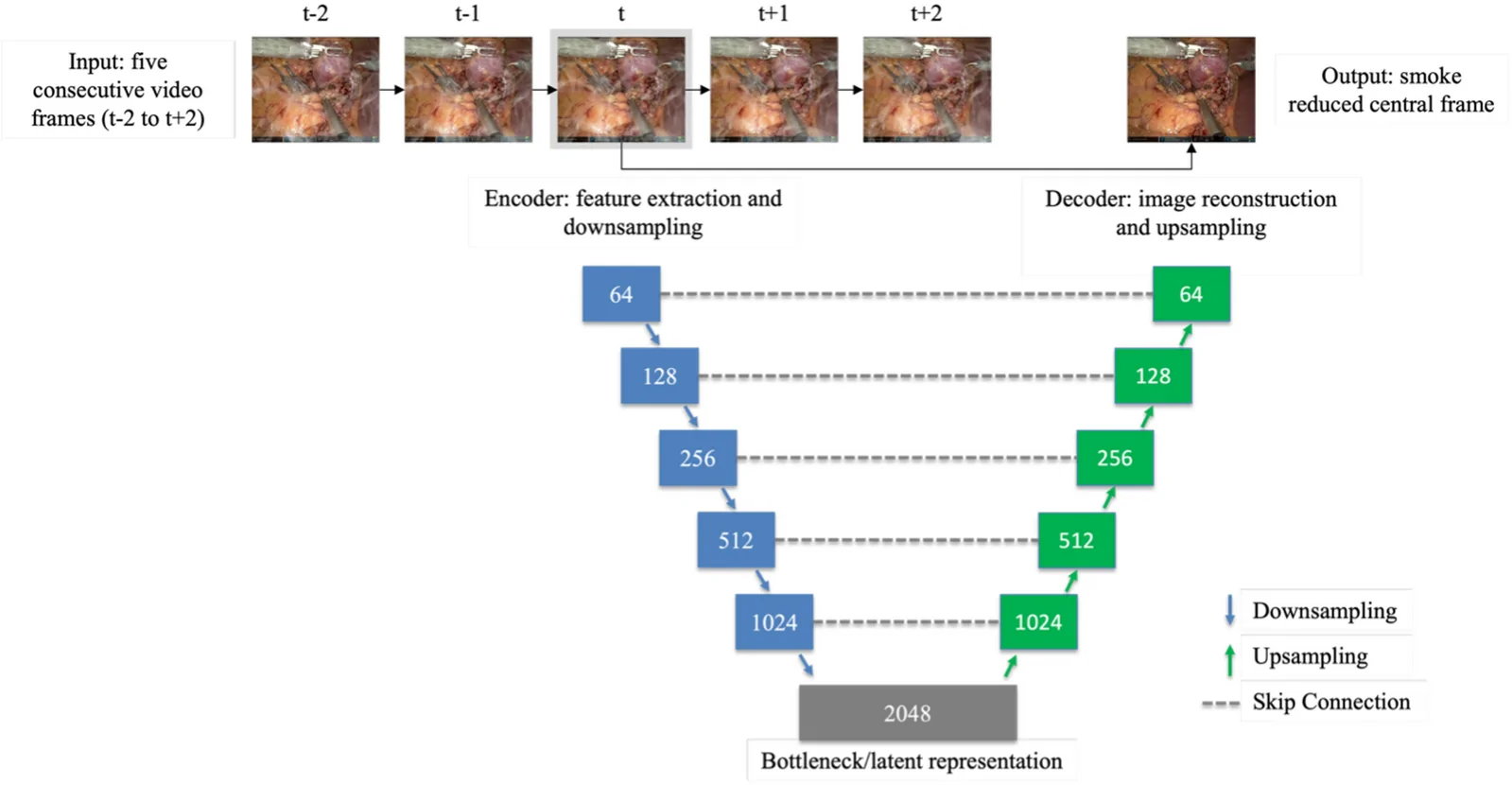
Simplified schematic of the temporally consistent 3D U-Net architecture. Five consecutive video frames (t − 2 to t + 2) are processed jointly. The encoder progressively extracts spatial and temporal features while reducing spatial resolution, whereas the decoder reconstructs the central output frame (t). Skip connections transfer high-resolution features from corresponding encoder stages to the decoder. Numbers indicate the number of feature channels. Figure adapted from [18]

The smoke removal algorithm evaluated in this study is based on the 3D U-Net architecture described by Janßen et al. [17]. The model uses five sequential endoscopic video frames as input to produce a single smoke-reduced output image corresponding to the temporal midpoint of the input sequence (see Fig. 1). This approach improves temporal consistency across consecutive frames. The algorithm was trained using a synthetic dataset created from recordings of robot-assisted partial nephrectomies. In this process, surgical smoke was digitally superimposed onto the original footage. Synthetic smoke was used because supervised training relies on pixel-accurate image pairs that show the same scene both with and without smoke. It was not possible to extract enough of such images in vivo, as there was not enough material available in which the same intraoperative moment was captured simultaneously under both conditions. Superimposing smoke onto smoke-free surgical images therefore provides the necessary reference for model training. In technical evaluation the PyTorch implementation achieved a higher SSIM and a lower warping error than a frame-wise 2D baseline, at an inference throughput of 16 frames per second compared with 38 frames per second for the baseline, measured on an Intel Core i9-13900 K CPU, 128 GB RAM and a GeForce RTX 4090 graphics card at a processing resolution of 720 × 1280 pixels. Detailed information on the model architecture and quantitative performance can be found elsewhere [17]. This study focuses on the clinical evaluation of the system.

### Participants

Study participants were recruited from the Department of Urology medical staff at UKSH Campus Kiel and Campus Lübeck. Eligible participants were attending urologic surgeons and surgical residents who had direct clinical experience with minimally invasive and/or robot-assisted urooncologic surgery. Participation was voluntary and anonymous. None of the twenty participants were involved in developing the algorithm, acquiring or selecting the video material, or analyzing the survey data. Additionally, none of them are authors of this manuscript. Similarly, none of the authors participated as raters. The following demographic information was collected: professional level (resident/attending) and experience with robotic or minimally invasive surgery (categorized as < 1 year, 1–3 years, 3–5 years, or > 5 years). Twenty clinicians completed the evaluation.

### Evaluation material

**Fig. 2:**
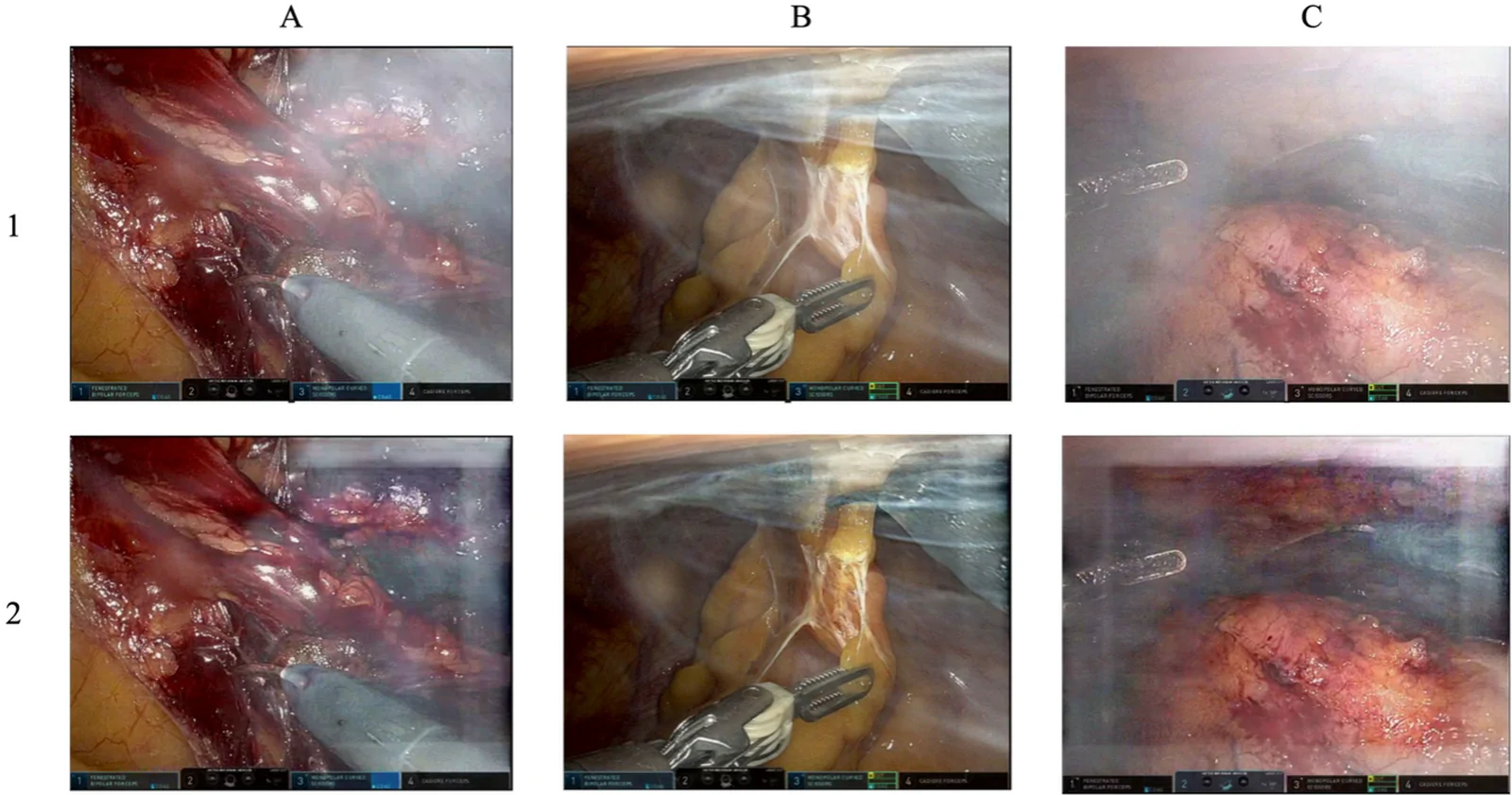
Representative examples of AI-based smoke removal in three intraoperative scenes with different smoke density (columns A–C). Row 1 shows the original smoke-affected frames, row 2 the same frames after processing with the temporally consistent 3D U-Net. Panels A2 and C2 show the rectangular border artifact reported by the evaluators. C2 additionally illustrates the residual haze remaining in the densest scene

The video material consisted of ten endoscopic clips recorded between September and December 2025 at the University Hospital Schleswig-Holstein, Campus Kiel, during four robot-assisted urological procedures performed on three patients. To ensure clinical relevance, the selected clips contained real surgical smoke rather than synthetic smoke. The small number of clips is due to the significant effort required to extract suitable video sequences from the raw footage. Following identification, potential clips were extracted, checked for identifying information and then AI-processed. The final selection was therefore made carefully to cover a variety of smoke densities and clinically relevant intraoperative scenarios (see Fig. 2 for an example), rather than to maximize the number of cases. The recordings included in the evaluation were completely separate from the video material used to train the model described in [17]. No patient contributed recordings to both the training and evaluation datasets. Each participant evaluated the corresponding video pairs, which consisted of the original smoke-obscured recording and its AI-processed version. The two clips in each pair were displayed side by side on the same screen, with the original shown on the left in a standardized order.

Each clip was ten seconds long. The footage was displayed in two-dimensional mode on a 24-inch monitor with a resolution of 1920 × 1080 pixels. Participants viewed the material individually, one pair at a time, and were not allowed to replay a pair. Both the original and AI-processed clips were displayed at a frame rate of 16 fps.

### Questionnaire instrument

Since no validated questionnaire for clinical assessment of AI-based smoke removal currently exists, a purpose-designed questionnaire was developed based on clinically relevant image quality domains. Full questionnaire is provided in the Supplementary Materials (translated to English from German language). After reviewing all ten clip pairs, each participant completed the questionnaire. All ratings reflect an overall assessment of the full set of clips, rather than evaluations of individual clips. The two response formats were selected for different purposes. Part 1 used an agreement scale applied to statements about the processed material. Several of these statements explicitly referenced the original recordings. Part 2 used a directional comparative scale with an explicit “equal” category anchored on the corresponding unprocessed clip.

The questionnaire comprised four sections:

#### Part 1: General impression

Seven statements about the AI-processed material were rated on a five-point Likert scale (1 = “strongly disagree”, 5 = “strongly agree”). The statements covered image clarity, recognizability of anatomical structures, realism, clinical usability, naturalness of color rendering, presence of disturbing artifacts, support of intraoperative orientation, and perceived helpfulness in the clinical routine.

#### Part 2: Direct before-and-after comparison

Four dimensions are rated as “worse”, “equal”, “slightly better” or “significantly better”: visibility of anatomical details, depth perception and spatial orientation, image stability, and realism of color rendering.

#### Part 3: Open-ended questions

Perceived benefits, artifacts noticed, surgical situations in which smoke removal would be most useful, and concerns regarding clinical application.

#### Part 4: Overall utility rating

“Very useful”, “Rather useful”, “Neutral”, “Less useful” or “Not useful.”

### Statistical analysis

Due to the exploratory nature of the study and the small sample size (*n* = 20), all analyses were purely descriptive. Responses were manually entered into a digital spreadsheet and verified against the original questionnaires before analysis. The responses were summarized as absolute counts and percentage frequencies for each response category and means (and standard deviations, where appropriate) were computed for each questionnaire item. The Supplementary Materials report item-level response distributions and descriptive statistics for all twelve closed questionnaire items in Table S2. Exact Clopper-Pearson 95% confidence intervals were calculated for response proportions using a two-sided approach. All twenty participants completed all twelve closed items, resulting in complete quantitative data and eliminating the need for imputation. The relevant denominator is provided whenever qualitative responses are expressed quantitatively. Responses to the open-ended questions were synthesized narratively. Inferential statistical testing and subgroup comparisons were not conducted. Statistical computations and visualizations were performed using R (version 4.5.2).

## Results

### Participant characteristics

Twenty urological clinicians from UKSH Campus Kiel and Campus Lübeck participated in the evaluation. All invited participants completed the survey, with a 100% response rate. Experience with minimally invasive or robotic surgery was distributed across all categories, with 35% reporting less than one year and 30% reporting more than five years of experience (Table 1). Due to the anonymous design and limited subgroup sizes, stratified analyses by experience level are not reported.

**Table 1:** Baseline characteristics of the participating clinicians (*n* = 20). MIS: minimally invasive surgery

Characteristic	*n*	%
Surgical resident	12	60%
Attending urologist	8	40%
Specialty: urology	20	100%
Experience MIS/robotic surgery: < 1 year	7	35%
1–3 years	4	20%
3–5 years	3	15%
> 5 years	6	30%

#### Part 1: General impression

**Fig. 3:**
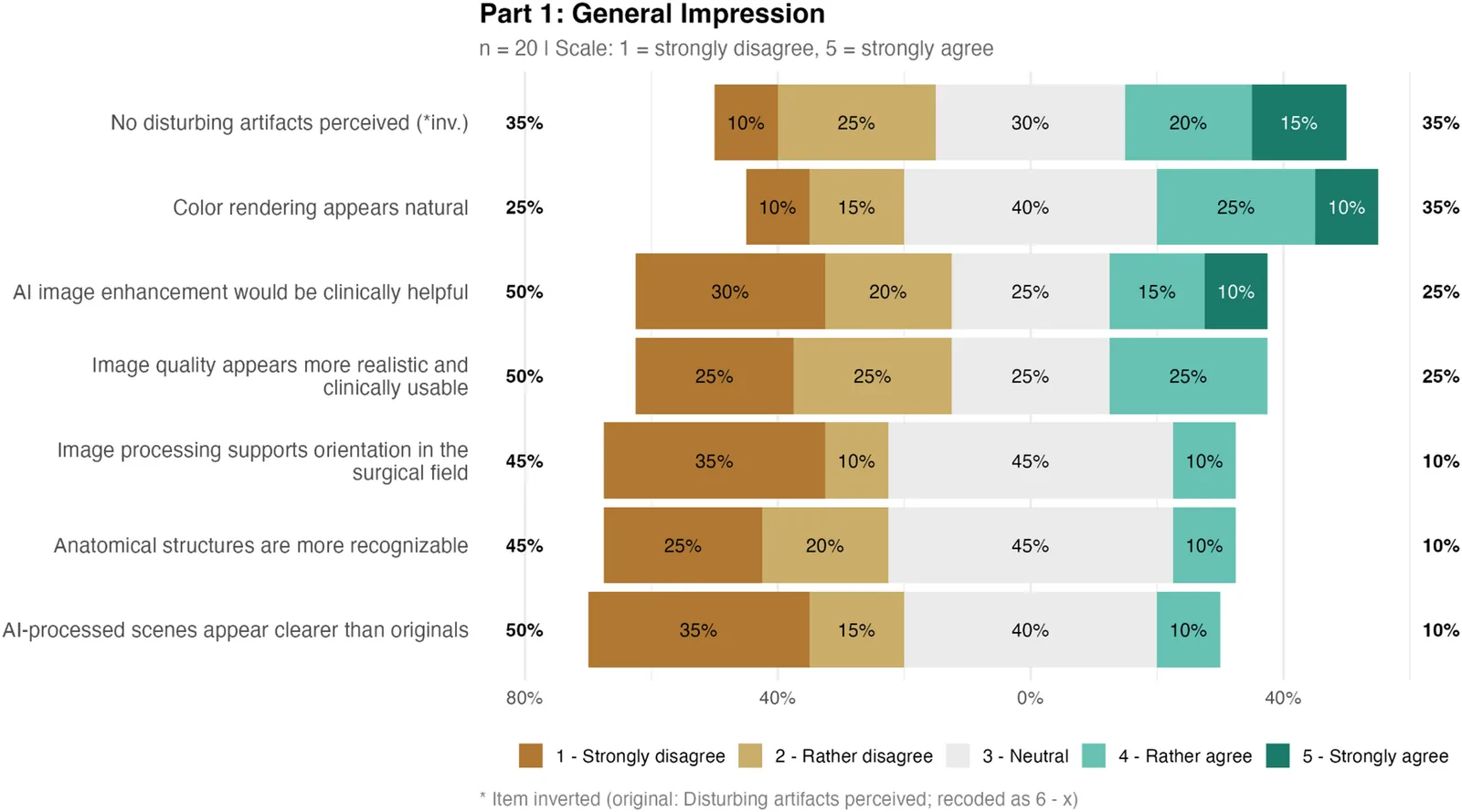
Diverging stacked bar chart for Part 1 (General Impression, *n* = 20). Amber tones (left) represent disagreement; teal tones (right) represent agreement. Bold percentages at each end indicate the total proportion of negative and positive responses. The artifact item is displayed in inverted form (“no disturbing artifacts perceived”) so that, for all items, responses to the right of the center line indicate favorable assessments

Figure 3 shows the results of the seven questions regarding overall impression. When asked if the AI-processed images appear clearer, 50% of participants disagreed or strongly disagreed. Participants predominantly rated the visibility of anatomical structures, assistance with orientation in the surgical field, realism, and clinical usefulness of image quality as neutral to negative. No participant strongly agreed with any of these four statements.

Color reproduction received the most positive evaluations: 35% of participants agreed or strongly agreed that the colors appeared natural. Regarding the perceived usefulness of AI image enhancement in everyday clinical practice, responses were mixed. Five participants (25%) agreed or strongly agreed, while ten (50%) disagreed or strongly disagreed. Seven participants (35%) agreed or strongly agreed that there were no distracting artifacts in the AI-processed material. Six participants (30%) were neutral on this point, and seven (35%) perceived distracting artifacts.

#### Part 2: Direct before-and-after comparison

**Fig. 4:**
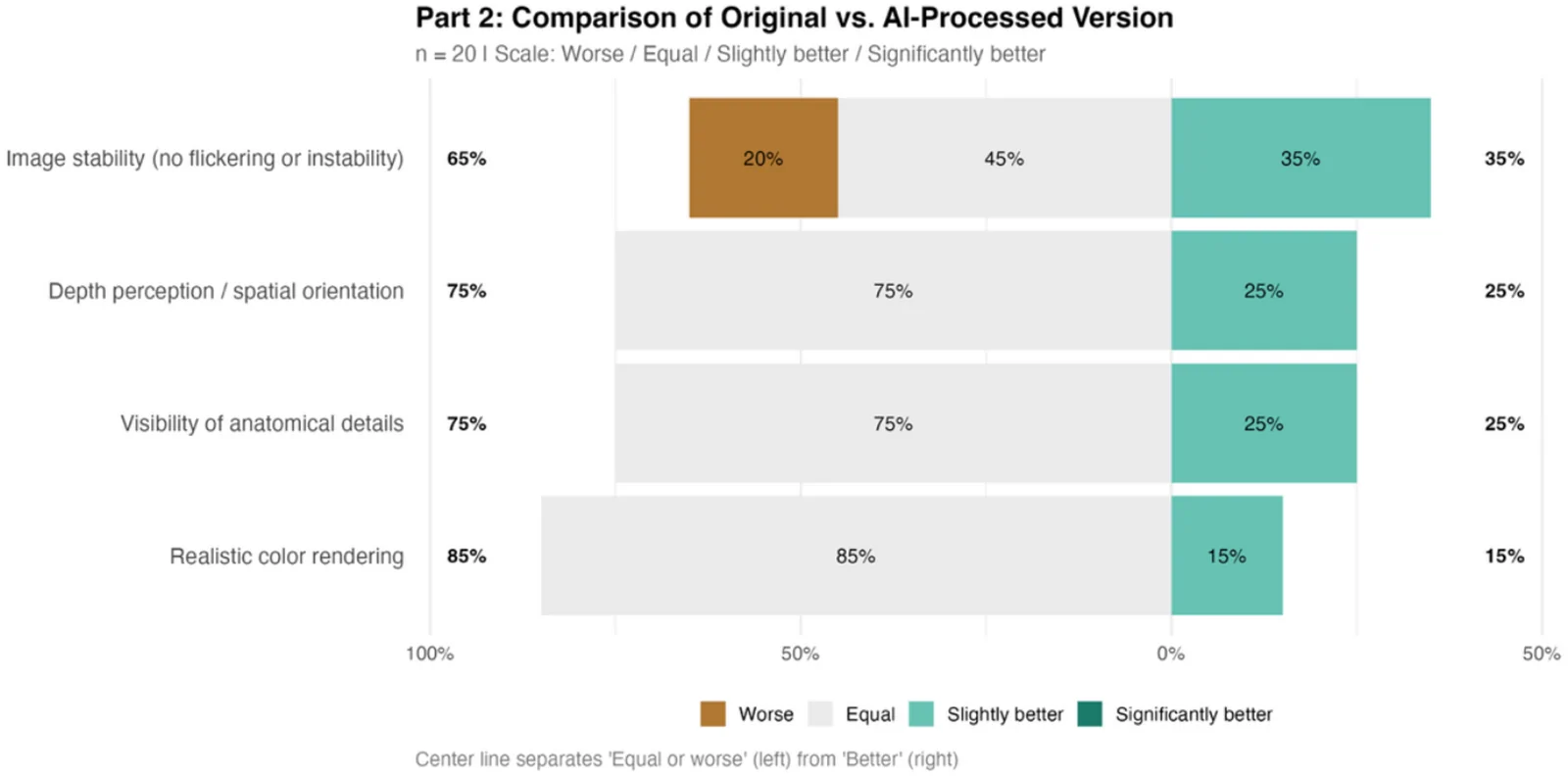
Diverging stacked bar chart for Part 2 (Before-and-After Comparison, *n* = 20). Left side (amber): rated “worse” than the original; right side (teal): rated better

Figure 4 shows the four image dimensions compared directly with the smoke-affected original footage. None of the participants selected the highest response category, “significantly better,” for any dimension. Visibility of anatomical details and depth perception were considered equal by 75% (15/20, 95% CI 50.9 to 91.3%) of participants and slightly better by 25% (5/20, 95% CI 8.7 to 49.1%). 85% (17/20, 95% CI 62.1 to 96.8%) rated realistic color rendering as equal, and 15% (3/20, 95% CI 3.2 to 37.9%) rated it as slightly better. For all three dimensions, no participant selected “worse” (0/20, exact 95% CI 0 to 16.8%). Image stability was the only dimension for which participants selected a worse rating, showing the greatest variation in responses. Overall, 35% (7/20, 95% CI 15.4 to 59.2%) rated the AI-processed video as slightly more stable, 45% (9/20, 95% CI 23.1 to 68.5%) considered it equally stable, and 20% (4/20, 95% CI 5.7 to 43.7%) thought the processed footage was less stable than the original.

#### Part 3: Qualitative feedback

The four open-ended questions were answered by nine, 14, 14, and 12 participants, respectively. Four participants described improved visibility of deeper tissue layers or a clearer view in otherwise poorly assessable situations, while four reported no relevant benefit. The reported artifacts varied and included gray, frame- or border-like structures at the edges of the image, horizontal bars or striped artifacts and flickering at the edge of the image (see Fig. 2A2, C2). Three participants also noted that smoke removal appeared to be incomplete in cases of dense smoke.

When asked in which surgical situations smoke removal would be most useful, the participants cited the coagulation of bleeding sites (five of 14), situations involving heavy smoke production (one), dissection near vessels (two), poorly assessable anatomical situations (two), working in confined spaces with poor ventilation (one) and nerve-sparing dissection during radical prostatectomy and retroperitoneal kidney surgery (one). Concerns regarding the clinical application of the technology centered on processing delays, image distortion, and the overall maturity of the technology. Six of the twelve participants who answered this question stated that they had no relevant concerns.

#### Part 4: Overall utility assessment

**Fig. 5:**
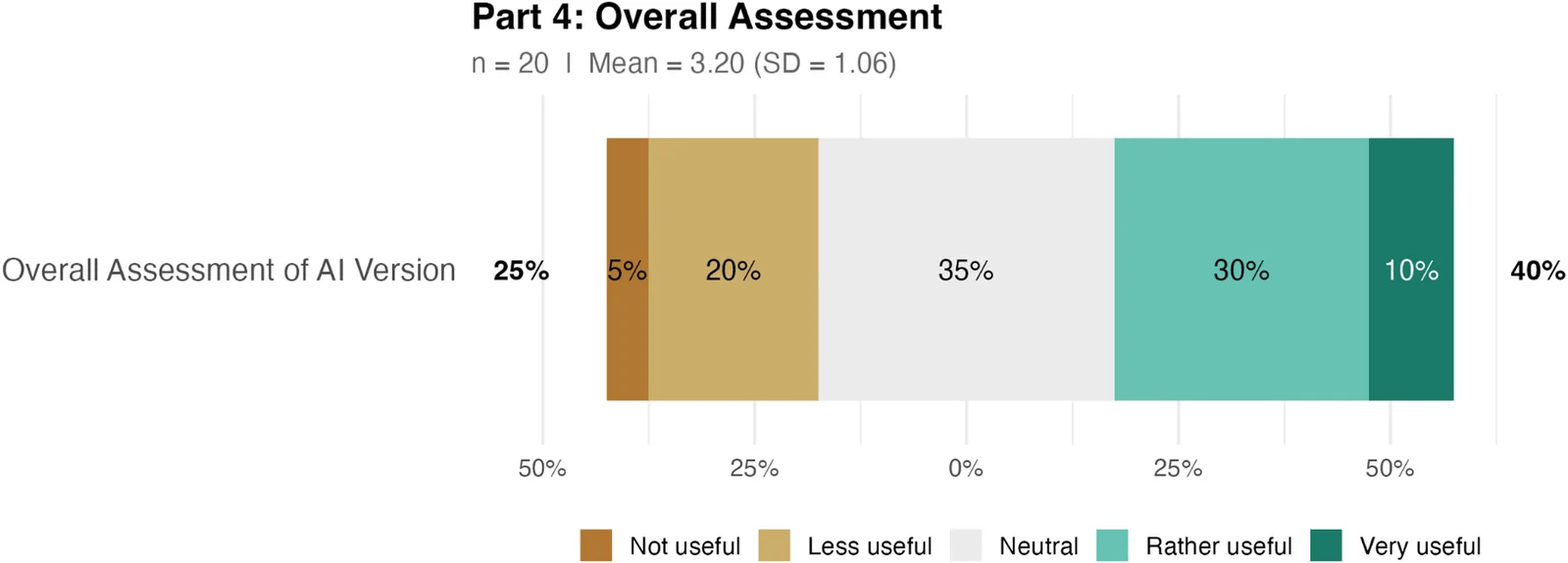
Diverging stacked bar chart for Part 4 (Overall Utility Assessment, *n* = 20). Left side (amber tones): not useful / less useful; right side (teal tones): rather / very useful. Bold percentages indicate the total proportion of negative (25%) and positive (40%) responses

Figure 5 shows the distribution of overall utility ratings. Two participants (10%) rated the AI system as “very useful,” six (30%) as “rather useful,” seven (35%) as “neutral,” four (20%) as “less useful,” and one (5%) as “not useful.” Overall, 40% rated the system as rather or very useful, 35% as neutral and 25% as less or not useful.

## Discussion

### Absence of perceived deterioration

In the direct comparison, none of the participants rated the visibility of anatomical details, depth perception, or color fidelity as worse after AI processing. 20% rated image stability as worse. However, with 0/20 worse ratings, the upper bound of the exact 95% confidence interval is 16.8% [19]. The study was not designed to demonstrate equivalence, and the results are further limited by the asymmetric scale in Part 2, which included one negative and two positive response categories.

The finding regarding image stability is particularly relevant, as improved temporal stability was an explicit goal of the underlying 3D U-Net architecture. A stable representation of the tissue and reliable orientation are of particular importance in robotic surgery, as visual information partially compensates for limited tactile feedback [20–22].

Parts 1 and 2 produced different response patterns. In Part 1, half of the participants disagreed that the processed videos appeared clearer. Recognizability of anatomical structures, orientation support, and realism were predominantly rated as neutral to negative. In Part 2, most ratings were equal to or slightly better. This difference partly reflects the response formats. Part 2 included an explicit “equal” category, whereas in Part 1, unchanged impressions could be interpreted as disagreement with positively phrased statements. The sections also assessed different constructs: Part 2 focused on specific image characteristics, while Part 1 focused on broader impressions and expected usefulness. Also, in Part 2 ratings of “equal” or “slightly better” do not indicate image quality that is comparable to a clear native operative view or sufficient for clinical use. Overall, AI processing did not produce a clear improvement in perception.

### Temporal consistency and artifact perception

Artifacts were a major concern. Seven participants (35%) reported disturbing artifacts, and 20% rated image stability as worse than the original. This contrasts with the technical evaluation, in which the 3D U-Net produced lower warping error and a higher SSIM than the 2D baseline [17]. Local artifacts may therefore be clinically noticeable despite having limited effects on global image quality metrics. This discrepancy has also been reported for other intraoperative AI systems [23, 24]. Since the present study only compared the 3D model with unprocessed footage, the clinical relevance of its technical advantage over a 2D baseline is unclear. Structured expert evaluation should therefore complement computational performance metrics during further development.

### Perceived clarity and orientation support

The reserved ratings for image clarity and orientation support may partly reflect the mismatch between training and evaluation data. The model was trained using synthetic smoke, whereas the present evaluation used authentic surgical smoke, which differs in optical density, spatial distribution, fine structure and turbulence [25]. This may contribute to incomplete smoke removal, particularly in dense smoke, as also noted in the free text responses and illustrated in Fig. 2C2.

Surgeons accustomed to high resolution three-dimensional intraoperative imaging may also apply strict internal standards when judging processed images [26, 27]. This may have contributed to the reserved Part 1 ratings, although perceptual anchoring cannot be established from the present data. Moreover, Part 1 was not purely absolute, as several items referred explicitly or implicitly to improvement over the original footage. Parts 1 and 2 should therefore be interpreted according to their different rating formats and reference points.

### Overall utility and clinical adoption potential

Overall utility of the tool was rated as “rather useful” or “very useful” by 40% of participants, “neutral” by 35%, and “less useful” or “not useful” by 25% (median 3.0, Table S2). These results indicate a predominantly neutral to moderately positive attitude, rather than strong enthusiasm or clear rejection. Since the evaluated system is in the early stages of research and has not been integrated into clinical practice yet, this level of acceptance is an appropriate basis for further technical refinement. The relatively low proportion of negative ratings suggests that participants are generally open to AI-assisted smoke removal if visible artifacts, latency, and incomplete smoke suppression can be minimized. This interpretation is consistent with current findings on intraoperative AI support in robotic surgery. Surgeons prefer applications that directly improve visual guidance, particularly in the detection of anatomical structures and the identification of risks [28]. Despite the generally positive attitude and trust in clinically validated AI tools, actual intraoperative use remains rare. This highlights the persistent gap between the perceived clinical potential of AI tools and their practical implementation.

The scenarios mentioned in the open-ended responses are characterized by a combination of heavy smoke, demanding surgical requirements, and limited visibility. AI-assisted smoke removal could therefore be most clinically relevant when it functions as a visual aid rather than a decision-making system. Future evaluation studies should stratify assessments by procedure type, smoke density, phase of surgery, and oncological complexity.

### Implications for clinical translation in oncological surgery

The ability to operate in real time depends on two different factors. The first of these is the inherent latency of the underlying architecture. Reconstructing frame t from frames t-2 to t + 2 requires two consecutive frames. At a recording rate of 30 frames per second, this equates to a delay of around 67 milliseconds, which does not accumulate. Given the ideal telesurgery threshold of less than 200 milliseconds, this would still be acceptable [29]. The second factor concerns throughput. At a frame rate of 16 frames per second, the model does not match the capture rate of the endoscopic stream, which is 30 frames per second. In this case, inference is the limiting factor. This can be optimized using the variant proposed in the underlying technical paper. This variant processes three frames and excludes future ones. Consequently, lookahead is eliminated and computational overhead is reduced, potentially degrading output quality [17].

The absence of detected deterioration alone does not justify continued development. Further work is warranted because the limitations identified in this study are concrete and can be solved technically. These limitations include a reproducible artifact profile, processing speed below the acquisition rate, and residual haze in scenes with dense smoke. A future confirmatory evaluation should aim to demonstrate a perceptible improvement in image clarity, rather than showing that deterioration has not occurred.

For further model optimization, the focus should be on the specific artifacts identified by the clinical reviewers, such as edge structures, streaks, and peripheral flickering. To enable seamless integration into the intraoperative video stream, processing speed must be improved. Additionally, the training dataset should be expanded to include authentic surgical smoke from various robot-assisted oncological procedures under different intraoperative conditions.

Whether these refinements will lead to clinically relevant benefits must be clarified in larger, prospective studies prior to clinical use.

### Limitations

Several limitations may have biased the results in a favorable direction. The study was not designed or powered to demonstrate equivalence or non-inferiority, and the wide confidence intervals reflect the limited precision of twenty raters. Part 2 used an asymmetric scale with one negative and two positive options, which may have favored positive responses. Future studies should use a balanced five-point scale with “equal” as the central category. Because no smoke-free reference was included, the processed output could not be compared with a clear native operative view.

The evaluation was neither blinded nor randomized. Clip pairs were shown in a fixed order, processed clips were visually recognizable because of edge artifacts (Fig. 2A2, C2), and participants were recruited from the same urology departments as the clinical investigators and knew that the algorithm had been developed within the participating institutions. These factors may have favored the processed material, so the observed ratings may represent an upper estimate of perceived benefit.

The questionnaire was purpose-designed, not pilot-tested, and lacks reliability data. Two Part 1 items addressed more than one concept. Because one questionnaire was completed after all ten clip pairs, ratings represent an overall impression and cannot be stratified by smoke density, procedure type or surgical phase. Only 9 to 14 participants answered the open-ended questions, which were summarized narratively. The small single-institution urology sample precluded meaningful subgroup or specialty analyses. A frame-wise 2D comparator was not included, the assessment was performed offline rather than intraoperatively, and the model was trained on synthetic smoke and remains too slow for live use.

## Conclusion

This pilot study provides a structured clinical evaluation of 3D U-Net based smoke removal in robotic urooncologic videos. Although the study was not designed to establish equivalence, none of the participants rated anatomical detail visibility, depth perception, or color fidelity as worse after AI processing, although confidence intervals were wide. 20% of participants rated image stability as worse, while perceived improvements in clarity and orientation remained limited. Expert feedback identified characteristic edge artifacts, streaks, flickering, and residual smoke as areas in need of technical refinement.

Further development should focus on training with authentic surgical smoke, artifact reduction, and real-time processing. Confirmatory multicenter studies should employ balanced response scales, randomized and concealed presentation, catch trials, clip-level ratings, and a frame-wise 2D comparator. This study highlights the discrepancy between technical performance metrics and surgeons’ perceptions and identifies specific requirements for the further clinical development of AI-based smoke removal.

## Supplementary Information

Below is the link to the electronic supplementary material.

Supplementary Material 1

## Data Availability

The data supporting the reported results are available from the corresponding author upon reasonable request. Raw questionnaire data are not publicly available due to privacy considerations. The intraoperative video recordings are not publicly available due to their inclusion of clinical patient data, which makes them subject to institutional and consent-related restrictions.
