# Supplementary material for: AI-based smoke removal in robotic urooncologic surgery: a clinical pilot study
Source: J Robot Surg. 2026 Sep 19;20(1):1007. doi: 10.1007/s11701-026-03951-z (PMC13589700; doi:10.1007/s11701-026-03951-z)
Supplement: Supplementary file 1 [file 11701_2026_3951_MOESM1_ESM.docx]

Table S1. Checklist for Reporting Of Survey Studies (CROSS).

| **Section / Topic** | **Item** | **Item description** | **Reported on page # / Comment** |
| --- | --- | --- | --- |
| **Title and abstract** | | | |
| Title and abstract | **1a** | State the word “survey” along with a commonly used term in title or abstract to introduce the study’s design. | 1–2. he term “survey” itself is not used in the title or abstract |
|  | **1b** | Provide an informative summary in the abstract, covering background, objectives, methods, findings/results, interpretation/discussion, and conclusions. | 1-2 |
| **Introduction** | | | |
| Background | **2** | Provide a background about the rationale of study, what has been previously done, and why this survey is needed. | 1-2 |
| Purpose / aim | **3** | Identify specific purposes, aims, goals, or objectives of the study. | 3 |
| **Methods** | | | |
| Study design | **4** | Specify the study design in the methods section with a commonly used term (e.g., cross-sectional or longitudinal). | 4 |
| Data collection methods | **5a** | Describe the questionnaire (e.g., number of sections, number of questions, number and names of instruments used). | 6 |
|  | **5b** | Describe all questionnaire instruments used to measure particular concepts. Report target population, reported validity and reliability information, scoring/classification procedure, and reference links (if any). | 6,12 |
|  | **5c** | Provide information on pretesting of the questionnaire, if performed (in the article or in an online supplement). Report the method of pretesting, number of times questionnaire was pre-tested, number and demographics of participants used for  pretesting, and the level of similarity of demographics between pre-testing participants and sample population. | 12 |
|  | **5d** | Questionnaire, if possible, should be fully provided (in the article, or as appendices or as an online supplement). | Supplementary Materials |
| Sample characteristics | **6a** | Describe the study population (i.e., background, locations, eligibility criteria for participant inclusion in survey, exclusion criteria). | 5 |
|  | **6b** | Describe the sampling techniques used (e.g., single stage or multistage sampling, simple random sampling, stratified sampling, cluster sampling, convenience sampling). Specify the locations of sample participants whenever clustered  sampling was applied. | 5 |
|  | **6c** | Provide information on sample size, along with details of sample size calculation. | 5,7 |
|  | **6d** | Describe how representative the sample is of the study population (or target population if possible), particularly for population-based surveys. | 12 |
| Survey administration | **7a** | Provide information on modes of questionnaire administration, including the type and number of contacts, the location where the survey was conducted (e.g., outpatient room or by use of online tools, such as SurveyMonkey). | 4,5 |
|  | **7b** | Provide information of survey’s time frame, such as periods of recruitment, exposure, and follow-up days. | 4 |
|  | **7c** | Provide information on the entry process:  –>For non-web-based surveys, provide approaches to minimize human error in  data entry.  –>For web-based surveys, provide approaches to prevent “multiple participation”  of participants. | 7 |
| Study preparation | **8** | Describe any preparation process before conducting the survey (e.g., interviewer training, advertising the survey). | N/A |
| Ethical considerations | **9a** | Provide information on ethical approval for the survey if obtained, including informed consent, institutional review board [IRB] approval, Helsinki declaration, and good clinical practice [GCP] declaration (as appropriate). | 13,14 |
|  | **9b** | Provide information about survey anonymity and confidentiality and mechanisms used to protect unauthorized access. | 5,14 |
| Statistical analysis | **10a** | Describe statistical methods and analytical approach. Report the statistical software used. | 7 |
|  | **10b** | Report any modification of variables used in the analysis, along with reference (if available). | 8 |
|  | **10c** | Report details about how missing data was handled. Include rate of missing items, missing data mechanism (i.e., missing completely at random [MCAR], missing at random [MAR] or missing not at random [MNAR]) and methods used to deal with missing data (e.g., multiple imputation). | 7 |
|  | **10d** | State how non-response error was addressed. | 7 |
|  | **10e** | For longitudinal surveys, state how loss to follow-up was addressed. | N/A |
|  | **10f** | Indicate whether weighting of items or propensity scores were used to adjust for non-representativeness. | N/A |
|  | **10g** | Describe any sensitivity analysis conducted. | N/A |
| **Results** | | | |
| Respondent characteristics | **11a** | Report numbers of individuals at each stage of the study. Consider using a flow diagram, if possible. | 7 |
|  | **11b** | Provide reasons for non-participation at each stage, if possible. | N/A |
|  | **11c** | Report response rate; present its definition or the formula used. | 7 |
|  | **11d** | Provide information to define unique visitors; report number of unique visitors with relevant proportions. | N/A |
| Descriptive results | **12** | Provide characteristics of study participants, information on potential confounders and assessed outcomes. | 7-10 |
| Main findings | **13a** | Give unadjusted estimates and, if applicable, adjusted estimates with 95% CIs and p-values. | 7-9, Supplementary Materials |
|  | **13b** | For multivariable analysis, provide model-building process, model-fit statistics, and model assumptions. | N/A |
|  | **13c** | Provide details about any sensitivity analysis; with substantial missing data, compare complete cases with imputed dataset. | N/A |
| **Discussion** | | | |
| Limitations | **14** | Discuss the limitations of the study, considering sources of potential biases and imprecisions, such as non-representativeness of sample, study design, important uncontrolled confounders. Discuss the limitations of the study, considering sources of potential biases and imprecisions, such as non-representativeness of sample, study design, important uncontrolled confounders. | 12-13 |
| Interpretation | **15** | Give a cautious overall interpretation of results, based on potential biases and imprecisions and suggest areas for future research. | 11-13 |
| Generalizability | **16** | Discuss the external validity of the results. | 12 |
| **Other sections** | | | |
| Role of funding source | **17** | State whether any funding organization has had any roles in the survey’s design, implementation, and analysis. | 13 |
| Conflict of interest | **18** | Declare any potential conflict of interest. | Declared in the submission system |
| Acknowledgements | **19** | Provide names of organizations/persons that are acknowledged along with their contribution to the research | Declared in the submission system |

| **Item** | **n** | **1** | **2** | **3** | **4** | **5** | **Median** | **Range** | **Mean** | **SD** | **Unfavorable, % (95% CI)** | **Favorable, % (95% CI)** |
| --- | --- | --- | --- | --- | --- | --- | --- | --- | --- | --- | --- | --- |
| **Part 1 - General impression of the AI-processed video** | | | | | | | | | | | | |
| 1.1 - Scenes appear clearer | 20 | 7 (35%) | 3 (15%) | 8 (40%) | 2 (10%) | 0 (0%) | 2.5 | 1–4 | 2.25 | 1.07 | 50% (27.2–72.8) | 10% (1.2–31.7) |
| 1.2 - Anatomical structures recognizable | 20 | 5 (25%) | 4 (20%) | 9 (45%) | 2 (10%) | 0 (0%) | 3.0 | 1–4 | 2.40 | 0.99 | 45% (23.1–68.5) | 10% (1.2–31.7) |
| 1.3 - Image quality realistic and clinically usable | 20 | 5 (25%) | 5 (25%) | 5 (25%) | 5 (25%) | 0 (0%) | 2.5 | 1–4 | 2.50 | 1.15 | 50% (27.2–72.8) | 25% (8.7–49.1) |
| 1.4 - Color rendering natural | 20 | 2 (10%) | 3 (15%) | 8 (40%) | 5 (25%) | 2 (10%) | 3.0 | 1–5 | 3.10 | 1.12 | 25% (8.7–49.1) | 35% (15.4–59.2) |
| 1.5 - No disturbing artifacts (inverted) | 20 | 2 (10%) | 5 (25%) | 6 (30%) | 4 (20%) | 3 (15%) | 3.0 | 1–5 | 3.05 | 1.23 | 35% (15.4–59.2) | 35% (15.4–59.2) |
| 1.6 - Supports intraoperative orientation | 20 | 7 (35%) | 2 (10%) | 9 (45%) | 2 (10%) | 0 (0%) | 3.0 | 1–4 | 2.30 | 1.08 | 45% (23.1–68.5) | 10% (1.2–31.7) |
| 1.7 - Helpful in clinical routine | 20 | 6 (30%) | 4 (20%) | 5 (25%) | 3 (15%) | 2 (10%) | 2.5 | 1–5 | 2.55 | 1.36 | 50% (27.2–72.8) | 25% (8.7–49.1) |
| **Part 2 - Direct before-and-after comparison with the original video** | | | | | | | | | | | | |
| 2.1 - Visibility of anatomical details | 20 | 0 (0%) | 15 (75%) | 5 (25%) | 0 (0%) | – | 2.0 | 2–3 | 2.25 | 0.44 | 0% (0.0–16.8) | 25% (8.7–49.1) |
| 2.2 - Depth perception / spatial orientation | 20 | 0 (0%) | 15 (75%) | 5 (25%) | 0 (0%) | – | 2.0 | 2–3 | 2.25 | 0.44 | 0% (0.0–16.8) | 25% (8.7–49.1) |
| 2.3 - Image stability (absence of flickering) | 20 | 4 (20%) | 9 (45%) | 7 (35%) | 0 (0%) | – | 2.0 | 1–3 | 2.15 | 0.75 | 20% (5.7–43.7) | 35% (15.4–59.2) |
| 2.4 - Realistic color rendering | 20 | 0 (0%) | 17 (85%) | 3 (15%) | 0 (0%) | – | 2.0 | 2–3 | 2.15 | 0.37 | 0% (0.0–16.8) | 15% (3.2–37.9) |
| **Part 4 - Overall assessment** | | | | | | | | | | | | |
| 4.1 - Overall utility of the system | 20 | 1 (5%) | 4 (20%) | 7 (35%) | 6 (30%) | 2 (10%) | 3.0 | 1–5 | 3.20 | 1.06 | 25% (8.7–49.1) | 40% (19.1–63.9) |

Table S2: Item-level response distributions and descriptive statistics are presented for all twelve closed-questionnaire items (n = 20; no missing data).

**Response categories**

**Part 1:** 1 = strongly disagree, 2 = disagree, 3 = neutral, 4 = agree, 5 = strongly agree.

**Part 2:** 1 = worse, 2 = equal, 3 = slightly better, 4 = significantly better; category 5 is not applicable (four-point scale).

**Part 4:** 1 = not useful, 2 = less useful, 3 = neutral, 4 = rather useful, 5 = very useful.

**Collapsed proportions:** for Parts 1 and 4, unfavorable = categories 1–2 and favorable = categories 4–5; for Part 2, unfavorable = “worse” (category 1) and favorable = “slightly” or “significantly better” (categories 3–4). Confidence intervals are exact (Clopper–Pearson), two-sided.

Values are absolute counts with row percentages in parentheses. Item 1.5 was phrased negatively in the questionnaire (“disturbing artifacts were perceived”) and is presented here in inverted form, so that higher values indicate a more favorable assessment for every Part 1 item. The Part 2 scale is asymmetric, offering one negative but two positive response options; this is addressed in the Limitations section of the main text.

**Supplementary Material - Questionnaire on the Evaluation of AI-Based Smoke Removal in Endoscopic Videos**

**General Information (voluntary, anonymous)**

- Level of experience: ☐ Resident ☐ Attending/board-certified specialist
- Specialty: ☐ Urology ☐ Surgery ☐ Gynecology ☐ Other: ___________
- Experience with robotic/minimally invasive surgery:

☐ < 1 year ☐ 1–3 years ☐ 3–5 years ☐ > 5 years

**Part 1: General Impression**

Please rate the following statements after viewing the presented video clips (1 = strongly disagree, 5 = strongly agree).

No.	Statement	1	2	3	4	5

1	The AI-processed scenes appear clearer than the original recordings.	☐	☐	☐	☐	☐

2	Anatomical structures (e.g., vessels, nerves, organ boundaries)	are easier to identify.	☐	☐	☐	☐	☐

3	The overall image quality appears more realistic and clinically usable.	☐	☐	☐	☐	☐

4	The coloring appears natural.	☐	☐	☐	☐	☐

5	I perceive disturbing artifacts or unnatural areas. (inverted scale)	☐	☐	☐	☐	☐

6	The image processing supports orientation within the surgical field.	☐	☐	☐	☐	☐

7	I would find such image enhancement helpful in everyday clinical practice.	☐	☐	☐	☐	☐

**Part 2: Comparison Before/After AI Processing**

Please rate the difference between the original and the AI-processed version.

**Aspect	Worse	Equal	Slightly better	Significantly better**

Visibility of anatomical details	☐	☐	☐	☐

Depth perception / spatial orientation	☐	☐	☐	☐

Image stability (no flickering or instability)	☐	☐	☐	☐

Realistic coloring	☐	☐	☐	☐

**Part 3: Open-Ended Questions**

1. Which aspects of the AI-processed videos do you find particularly helpful?

Answer: ____________________________________________

2. Which artifacts or disturbances did you notice?

Answer: ____________________________________________

3. In which surgical situations do you consider smoke removal particularly useful?

Answer: ____________________________________________

4. Do you have any concerns regarding clinical application (e.g., trust, color changes, latency)?

Answer: ____________________________________________

**Part 4: Overall Assessment**

Please mark one overall rating:

☐ Very useful ☐ Rather useful ☐ Neutral ☐ Less useful ☐ Not useful
